# Thinking Forward: The Quicksand of Appeasing the Food Industry

**DOI:** 10.1371/journal.pmed.1001254

**Published:** 2012-07-03

**Authors:** Kelly D. Brownell

**Affiliations:** Rudd Center for Food Policy and Obesity, Yale University, New Haven, Connecticut, United States of America

## Abstract

In an article that forms part of the *PLoS Medicine* series on Big Food, Kelly Brownell offers a perspective on engaging with the food industry, and argues that governments and the public health community should be working for regulation, not collaboration.

*This article was commissioned for the* PLoS Medicine *series on Big Food that examines the activities and influence of the food and beverage industry in the health arena.*

It is an important time to reflect on the ways that the public and global health communities can engage with the food industry. There are divergent views [1]. Many political bodies, foundations, and scientists believe that working collaboratively with the food industry is the path for change. The assumption is that this industry is somehow different than others, and that because people must eat, the industry is here to stay, and like it or not, working with them is the only solution.

Based on my 30 years of experience in the public health and policy sectors, I believe this position is a trap. When the history of the world's attempt to address obesity is written, the greatest failure may be collaboration with and appeasement of the food industry. I expect history will look back with dismay on the celebration of baby steps industry takes (such as public–private partnerships with health organizations, “healthy eating” campaigns, and corporate social responsibility initiatives) while it fights viciously against meaningful change (such as limits on marketing, taxes on products such as sugared beverages, and regulation of nutritional labeling).

The obesity problem has industry's attention, and they are doing things. The question is whether these things are meaningful or are the predictable behavior of an industry under threat and are designed to stop rather than support public health efforts. The soft drink industry gave the Children's Hospital of Philadelphia a US$10 million gift—at a critical time the city of Philadelphia was considering a soda tax. Such public-sector interaction with industry could be predicted to undermine public health goals and protect industry interests [2]–[6].

The food industry has had plenty of time to prove itself trustworthy. It has been in high gear, making promises to behave better, but their minor progress creates an impression of change while larger attempts to subvert the agenda carry on. Witness the massive resistance against soda taxes in the United States [7] and the wholesale attack of marketing standards proposed by the Interagency Working Group (e.g., [8]). Worst perhaps is the issue of marketing food to children. The industry launched the Children's Food and Beverage Advertising Initiative designed to “…shift the mix of foods advertised to children under 12 to encourage healthier dietary choices and healthy lifestyles” [9]. Objective reports, however, have shown a tidal wave of marketing of calorie-dense, nutrient-poor foods to children, and if any change is occurring, marketing is on the increase [10]–[13].

Companies boast of introducing healthier options, and at least one report cites this as evidence that market forces (e.g., consumer demand for better foods) will be the best motivator for companies to change [14]. But introducing healthier processed foods does not mean unhealthy foods will be supplanted, and might simply represent the addition of more calories to the food supply. Furthermore, the companies have not promised to sell less junk food. Quite the contrary; they now offer ever larger burgers and portions, introduce ever more categories of sugared beverages (sports drinks, energy drinks, and vitamin waters), find ever more creative ways of marketing foods to vulnerable populations (e.g., children), and increasingly engage in promotion of unhealthy foods in developing countries [1],[15],[16].

The food industry, like all industries, plays by certain rules—it must defend its core practices against all threats, produce short-term earnings, and in do doing, sell more food [2],[17]. If it distorts science, creates front groups to do its bidding, compromises scientists, professional organizations, and community groups with contributions, blocks needed public health policies in the service of their goals, or engages in other tactics in “the corporate playbook” [3],[18], this is what is takes to protect business as usual.

The parallel scenario most often used to justify collaboration with industry is tobacco. Often heard is that “people don't have to smoke, but they must eat” and that “the tobacco industry was simple—just a few companies and one product—but food is much more complex” [3]. Tobacco is an interesting parallel [3],[19], but is by no means the only one. A world economic crisis was fueled in part by too little oversight of financial institutions, but we all need banks. Requiring air bags in cars was stalled for years by the auto industry, but we need cars.

An emerging area in need of scrutiny is the food industry's attempts to create foods engineered in ways that thwart the human body's ability to regulate calorie intake and weight. Whether overconsumption is a consequence simply of hyperpalatability brought about by extreme processing [15] and/or an addictive process [20],[21], overconsumption is a predictable consequence of the current food environment. The arresting reality is that companies must sell less food if the population is to lose weight, and this pits the fundamental purpose of the food industry against public health goals.

We need food, but the obesity crisis is made worse by the way industry formulates and markets its products. The food industry, like other industries must be regulated to prevent excesses and to protect the public good. Left to regulate itself, industry has the opportunity, if not the mandate from shareholders, to sell more products irrespective of their impact on consumers. Government, foundations, and other powerful institutions should be working for regulation, not collaboration.

If history is to look back positively on current times, the future must bring several things. Respectful dialogue with industry is desirable, and to the extent industry will make voluntary changes that inch us forward, the public good will be served. But there must be recognition that this will bring small victories only and that to take the obesity problem seriously will require courage, leaders who will not back down in the face of harsh industry tactics, and regulation with purpose.
